# The Prebiotic Effect of an Organic Acid Mixture on *Faecalibacterium prausnitzii* Metabolism and Its Anti-Pathogenic Role against *Vibrio parahaemolyticus* in Shrimp

**DOI:** 10.3390/biology12010057

**Published:** 2022-12-29

**Authors:** Eugenia Butucel, Igori Balta, David McCleery, Adela Marcu, Ducu Stef, Ioan Pet, Todd Callaway, Lavinia Stef, Nicolae Corcionivoschi

**Affiliations:** 1 Bacteriology Branch, Veterinary Sciences Division, Agri-Food and Biosciences Institute, Belfast BT4 3SD, Northern Ireland, UK; 2 Faculty of Bioengineering of Animal Resources, University of Life Sciences King Mihai I from Timisoara, 300645 Timisoara, Romania; 3 Faculty of Food Engineering, University of Life Sciences King Mihai I from Timisoara, 300645 Timisoara, Romania; 4 Department of Animal and Dairy Science, University of Georgia, Athens, GA 30602, USA

**Keywords:** *Faecalibacterium prausnitzii*, *Vibrio parahaemolyticus*, prebiotics, probiotics, bacterial growth, butyrate, virulence, primary epithelial cells

## Abstract

**Simple Summary:**

The harsh aquatic environment makes the use of dietary probiotics not cost-effective and with low efficacy due to their inability to reach the gut. Therefore, supporting the growth of commensal probiotic bacteria, in aquatic animals, represents an efficient strategy to improve and maintain their gut health. The commensal probiotic, *Faecalibacterium prausnitzii*, is well known for its association with improved gut health in humans, farm animals and crustaceans. As such, increasing its abundance in the gut is of major interest and, moreover, identification of interventions able to support its growth is necessary. Undoubtedly, organic compounds, such as mixtures of organic acids, have a role in improving gut health and supporting the gut microbiota in either fighting disease or improving digestion. With this study, we aimed to further understand the biological mechanisms by which *F. prausnitzii* inhibits pathogenic bacteria, in the presence of organic acids. Furthermore, we have also investigated the organic acid ability to stimulate bacterial growth in vitro and ex vivo. Herein we show that a mixture of organic acids, AuraAqua (Aq) leads to improved *F. prausnitzii* growth and butyrate production which consequently reduces *V. parahaemolyticus* infection of shrimp gut primary epithelial cells.

**Abstract:**

Increasing the abundance of probiotic bacteria in the gut requires either direct dietary supplementation or the inclusion of feed additives able to support the growth of beneficial commensal bacteria. In crustaceans, the increased presence of probiotic-like bacteria in the gut, including of *Faecalibacterium prausnitzii* (*F. prausnitzii*), will guarantee a positive health status and a gut environment that will ensure enhanced performance. The aim of this study was to investigate if a mixture of organic acids, AuraAqua (Aq) can stimulate the growth and the anti-pathogenic efficacy of *F. prausnitzii* through a combination of in vitro and ex vivo models. The results showed that 0.5% Aq was able to improve the growth rate of *F. prausnitzii* in vitro and in an ex vivo shrimp gut model. Moreover, we were able to demonstrate that Aq increases butyrate production and cellulose degradation in culture or in the shrimp gut model. The growth-stimulating effect of Aq also led to an improved and anti-pathogenic effect against *Vibrio parahaemolyticus* in a co-culture experiment with shrimp gut primary epithelial cells (SGP). In conclusion, our work demonstrates that Aq can stimulate the growth of *F. prausnitzii*, increase the production of short-chain fatty acid (SCFA) butyrate, improve substrate digestion, and prevent *V. parahaemolyticus* invasion of SGP cells.

## 1. Introduction

Shrimp growth and survival are significantly reduced by specific bacterial pathogens and the current treatment approaches, including antibiotics for disease prevention, are strongly discouraged due to their detrimental impact on consumers [1]. As previously described, dietary supplementation of probiotics is known to enhance growth performance, immune responses, and resistance to pathogen infections in white shrimp [2]. They are widely considered an efficient strategy to reduce antibiotic usage in crustaceans, however, they are currently used with limited success given the harsh conditions in aquatic environments [3]. This limitation reduces the possibility of using such interventions against the toxin-producing bacterium, *V. parahaemolyticus*, responsible for significant losses in shrimp farming by causing acute hepatopancreatic necrosis disease (AHPND) [4]. Under these circumstances, the identification of interventions able to stimulate the activity of commensal probiotic-like bacteria seems the most plausible solution to control the activity of bacterial pathogens such as *V. parahaemolyticus* [5,6]. The approach of using dietary supplements to stimulate the commensal bacteria, to improve performance and disease resistance in shrimp, was recently described when two commensal mannanase-secreting bacteria (Man26 and Man122) were able to increase mannan digestion and potentially improve absorption and utilization of feedstuffs [7]. Stimulating the growth of probiotic bacteria (e.g., *F. prausnitzii*) and increase butyrate production was previously achieved-achieved with oligosaccharides and resulted in improved gut health and increased resistance to infections [8]. Moreover, once their growth was enhanced in the gut, probiotics (e.g., *Lactobacillus plantarum* Ep-M17 of fish origin) can improve not only the above-mentioned performance parameters in shrimp, but also protected against *V. parahaemolyticus* E1 infection [9].

*Faecalibacterium prausnitzii* (*F. prausnitzii*), a Gram-positive, oxygen-sensitive and butyrate-producer commensal bacterium, is well-known for its therapeutic role in inflammatory diseases, the so-called next-generation probiotic [10]. Interestingly, the levels of *F. prausnitzii* are significantly lower in shrimp affected by the acute hepatopancreatic necrosis disease (AHPND), compared to healthy shrimp, suggesting that an increased presence of *F. prausnitzii* might help the organism fight against infection [11]. This observation was also noted in porcine caeca, where *Salmonella* Typhimurium infections were reduced through dietary interventions resulting in higher levels of *F. prausnitzii* [12], particularly because *Salmonella* spp., infections are associated with low levels of this probiotic [13].

A mixture of organic acids and plant extracts have been extensively investigated for their role in preventing pathogen attachment [14] and invasion of epithelial cells in vitro [15], for improving the host immune system [16], for downregulating the expression of bacterial virulence genes and for enhancing gut health and animal performance in vivo [17]. Some of these effects are the direct result of enhanced probiotic presence [18], leading to pathogen exclusion and decreased inflammation in vivo [19]. In humans, organic compounds (polyphenols) can induce changes in gut microbiota, and directly increasing the numbers of *F. prausnitzii* in metabolic syndrome patients is considered an effective strategy for managing metabolic diseases [20].

It is then clear that organic acids are a very efficient tool to improve gut health and protect against infections, with enhanced activity when used as blends rather than as individual components [21]. On that basis, with the current study, we aimed to extend these observations to the probiotic bacterium *F. prausnitzii*, known for its involvement in enhancing gut health and the release of the short-chain fatty acid butyrate in shrimp [11]. Moreover, we sought to investigate if the positive impact on *F. prausnitzii*, translates into a prevention strategy against *V. parahaemolyticus* infection of shrimp primary epithelial cells.

## 2. Materials and Methods

### 2.1. F. prausnitzii Growth Performance in the Presence of AuraAqua

*F. prausnitzii* (DSM107838) strain was grown under anaerobic conditions at 37 °C on CDM1 (chemically defined medium) minimal medium [22]. *F. prausnitzii* agar-grown colonies were used to inoculate 100 mL of CDM1 minimal medium broth and cultured anaerobically at 37 °C for 24 h. During growth, samples were taken for optical density (OD_600_) measurements using a microplate reader (FluoStar Omega, Premier Scientific, Belfast, UK). To establish the impact of AuraAqua (Aq) on *F. prausnitzii*, concentrations of 0.2%, 0.5%, 1% and 2% Aq were included in the growth media during the 24 h or growth. The organic acid mixture, Aq contains 5% maltodextrin, 1% sodium chloride, 42% citric acid, 18% sodium citrate, 10% silica, 12% malic acid, 9% citrus extract, and 3% olive extract (*w*/*w*). The raw materials were supplied by Bio-Science Nutrition Ireland Co. (Limerick, Ireland).

### 2.2. In Vitro Shrimp Gut Models

The In vitro gut model system was conducted in three replicates as previously described [23]. This model was performed on un-spiked faeces when *F. prausnitzii* by RT-PCR and on spiked faeces when the bacterium was quantified as CFU (colony forming units) viable counts as described further in the methods. For the control experiment, 1000 mL of basal growth medium (4 g/L NaHCO_3_; 0.5 g/L K_2_HPO_4_, 0.5 g/L KH_2_PO_4_, 0.09 g/L MgSO_4_, 0.09 g/L CaCl_2_, 30 g/L NaCl, 0.5 mg/L resazurin and 10 mg/L hemin) was inoculated with 1 mL of shrimp faeces and incubated anaerobically at 30 °C for 24 h. Additionally, the four experimental groups included 0.2%, 0.5%, 1% and 2% Aq from the beginning of the experiment. The shrimp faeces were collected from 30 *P. vannamei* locally sourced shrimps, pooled, and diluted in basal growth medium (*w*/*v*). During the 24 h growth interval samples were collected at 0, 3, 6, 12 and 24 h for microbial enumeration and DNA extraction. In a second model, to ensure that the butyrate detected is accounted to *F. prausnitzii*, the gut model was modified such as the shrimp faeces were also irradiated to eliminate other viable bacteria. The irradiated faeces were spiked with 10^3^ *F. prausnitzii* and incubated further for 24 h anaerobically at 30 °C, followed by sampling for bacterium released butyrate.

### 2.3. Quantification of F. prausnitzii in the Shrimp Gut Model by RT-PCR and by Viable Counts

Faecal gDNA was extracted using the QIAamp DNA Stool Mini Kit (Qiagen, Manchester, UK) according to the manufacturer’s instructions and stored at −20 °C until use. Quantification of *F. prausnitzii* was performed as previously described [24]. The 16S rRNA primers used for *F. prausnitzii* quantification were 16SF ggaggaagaaggtcttcgg and 16SR aattccgcctacctctgcact [25]. For detection of universal bacteria, the actcctacgggaggcagcagt (F) and gtattaccgcggctgctggcac (R) primers were used [26]. The endpoint PCR and the RT-PCR were performed as previously described [27]. Briefly, the *F. prausnitzii* 16S rRNA gene copies were quantified using the LightCycler 96 (Roche, Burgess Hill, UK) and standard curves build with 10-fold serial dilutions of amplified *F. prausnitzii* rRNA genes. The relative abundance of *F. prausnitzii* 16S rRNA gene copies was calculated Relative abundance of target bacteria species with respect to the abundance of total bacteria: 2^−ΔCt^ = 2^−(Ct of target bacteria − Ct of total bacteria)^. To verify the PCR efficiency, standard curves were generated by 10-fold dilutions of bacterial DNA for all primer sets. The limit of detection of qPCR assays was established at 10–100 copies. When irradiated faeces were used in the shrimp gut model the growth of *F. prausnitzii* was measured by viable counts. Duplicate ten-fold serial dilutions of the bacterial solutions were made and four 50 μL of each dilution was plated in spots on anaerobic BHI agar and then incubated anaerobically for 48 h at 37 °C. Spots with between 10 and 100 colonies were counted and the CFU were calculated. The experiment was performed in triplicate.

### 2.4. Butyrate Measurement during F. prausnitzii Growth in the Presence of AuraAqua

Butyrate measurements were performed either in culture supernatants or in the in vitro gut model as described above. In the case of the in vitro gut model, butyrate was not measured in non-irradiated samples due to the presence of other microorganisms potentially also producing butyrate. To quantify the butyrate production in the in vitro gut model, the spiked faecal samples were homogenised and stored at −20 °C until processed and prior to measurement were diluted (1:4) in sterile water and homogenised in a stomacher (Seward, Premier, Scientific, Portsmouth, UK). Butyrate was also measured (0, 3, 6, 12, 24 h) in supernatants of the control sample (no Aq) and in the in vitro experimental growth cultures containing 0.2%, 0.5%, 1% and 2% Aq. All experiments and measurements were performed in triplicates. Butyrate production was measured by GC-MS (SCION-456-GC) as previously described [28].

### 2.5. In Vitro Butyrate Production in the Presence of a Cellulose Substrate

To quantify if the growth advantage gained by *F. prausnitzii* in the presence of 0.5% Aq, we have used the cellulose digestion method based on the principle of weight loss of block of pure cellulose during culture using CDM1 minimal media. When the bacterial density reached a concentration of 10^3^ (time 0), the cellulose membrane was inserted in the culture containers and further incubated in anaerobic conditions for 24 h. After 24 h the cellulose block was washed gently with distilled water, dried and weighed. The weight loss of the cellulose block was expressed as percentage of the initial weight. Butyrate production and *F. prausnitzii* growth profile was determined as described above. The experiments were conducted in triplicate and designed as follows: Control A, in the presence of *F. prausnitzii* only; Control B—media only; Control C—media only and 0.5% Aq; Experiment—*F. prausnitzii* and 0.5% Aq.

### 2.6. Infection of Shrimp Primary Cells (SGP) with V. parahaemolyticus in the Presence F. prausnitzii

The shrimp primary cells (SGP) were prepared from *P. vannamei* gut tissue samples as previously described [14]. Briefly, the gut tissue was cut into small pieces and gently washed twice by centrifugation (5 min, 150× *g*). Five ml 0.25% trypsin at pH 7.4 at room temperature was added for 30–60 min and stirred on a magnetic stirrer, washed twice and cells were put into a 25 cm plastic culture flask with growth medium. Cells were further cultured at 28 °C in 24 well plates (Analab, Lisburn, UK) supplemented with 0.1% DMSO (Thermo-Fisher, UK) and 20% foetal bovine serum (FBS), 100 µg penicillin, 8% shrimp head extract, 6% salt solution, 20 ng epidermal growth factor (Sigma-Aldrich, Gillingham, UK) and 10 U/mL human recombinant interleukin 2 (Sigma-Aldrich, Gillingham, UK). The *Vibrio parahaemolyticus* A3 (origin Vietnam) strains were kindly donated by Kim Orth from the Department of Molecular Biology, University of Texas Southwestern Medical Center, Dallas, TX, USA. The strain was routinely cultured in marine Luria–Bertani (MLB) and incubated aerobically at 37 °C for 24 h. *F. prausnitzii* cultures were grown in CDM1 media, containing 0.5% Aq, for 12 h under anaerobic conditions and harvested by centrifugation for 5 min at 3000 rpm and resuspended in CDM1 media at an OD600 of ∼0.4. A volume of 300 μL of *F. prausnitzii* in tissue culture medium was used to colonise the SGP cells for an initial period of 12 h. The SGP cell culture media was then replaced with 300 μL cell culture media containing *V. parahaemolyticus* A3 (OD_600_ of ∼0.15, MOI 100). Infected cells were further incubated for 24 h. To minimize changes in the pH medium on cells was replaced with fresh medium at the 12th hours following infection. Colonisation with *F. prausnitzii* did not affect the viability of SGP cells, as identified by trypan blue assays. After the infection period, a total association and invasion assay was performed to quantify bound *V. parahaemolyticus* A3 and *F. prausnitzii* by CFU as described above. As controls, *F. prausnitzii* colonised SGP cells in the absence of Aq (Control 1) and non-colonised SGP cells (Control 2) were also infected with *V. parahaeomolyticus* A3. To confirm the efficacy of Aq exposed in reducing the adhesion and invasion abilities of *V. parahaeomolyticus* A3 after infection, the infection media was removed, and the infected monolayers were washed three times with the tissue culture media. The infected cells were then incubated with tissue culture media containing gentamicin (100 µg/mL). To expose the internalised bacteria prior to total lysis without gentamicin inclusion, but, instead, 0.1% Triton X was included to reveal the total bacterial adhesion.

### 2.7. RT-PCR for Butyryl-CoA Transferase Quantification

The butyryl-CoA transferase gen- positive bacteria were measured as previously described [29]. The genomic DNA was extracted from 250 mL of bacterial culture as described in Section 2.3. Butyryl-CoA transferase gene-positive bacteria were amplified using the following primers BCoATsxrF: 5′-gcigaicatttcacitggaaywsitggcayatg and BCoATscrR: 5′-cctgcctttgcaatrtciacraangc-3′ [30]. For RT-PCR we have used the following conditions: hold 95 °C (5 min), 40 cycles of 95 °C (30 s), 53 °C (30 s), and 72 °C (30 s). Relative quantification of PCR products was determined with the 2^∆∆Ct^ method. The 16 s rDNA of total bacteria was used as a reference gene.

### 2.8. Statistical Analysis

Statistical analyses were performed using GraphPad Prism 9 software (Dotmatics, Boston, MA, USA). Significance was assigned at *p*-values  <  0.05 following estimations using Student’s *t*-test. Experiments were conducted on at least three separate occasions in triplicates. The Student *t*-test was used to estimate statistical significance. A *p* value of < 0.05 was considered significant.

## 3. Results

### 3.1. The In Vitro Effect of AuraAqua (Aq) on F. prausnitzii Growth in Minimal Media

**Figure 1:**
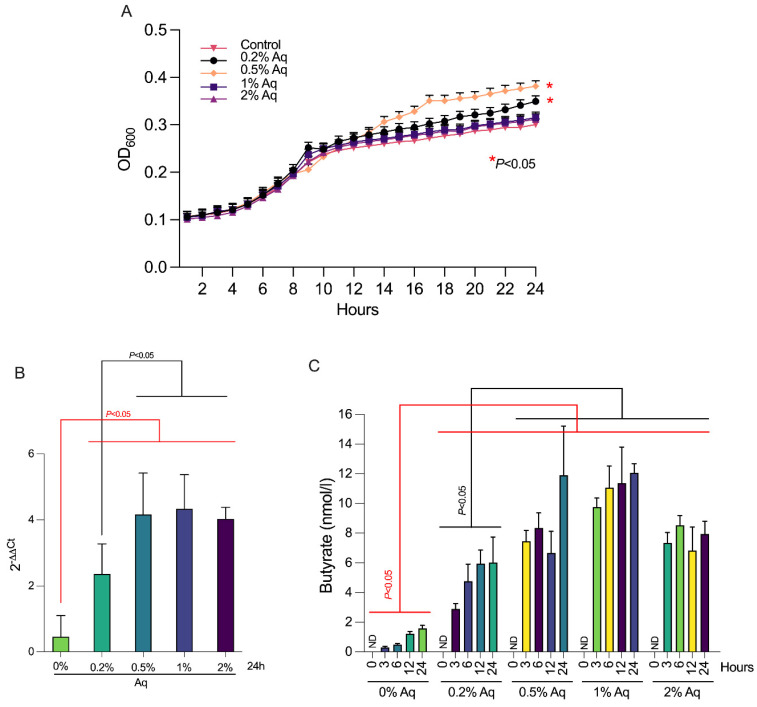
The impact of Aq on *F. prausnitzii* growth and butyrate production at concentrations of 0.2, 0.5, 1, and 2% to investigate the growth stimulatory effect. The impact on growth was quantified by measuring the absorbance at 600 nm for 24 h at 2 h intervals. Panel (**A**), butyryl-CoA transferase (*But*) gene was quantified from the total bacteria in minimal media at 24 h. The *However,* gene was quantified using 2^ΔΔCT^ analysis and results are expressed as mean SEM (standard error of mean). Student *t* test was used to identify significance at values below *p* < 0.05 (Panel (**B**)). Panel (**C**) shows the amount of butyrate released by *F. prausnitzii* in minimal media at 0.2%, 0.5%, 1% and 2% Aq and at intervals of 0, 3, 6, 12 and 24 h. All experiments were performed triplicate and each measurement was performed from three separate samples. A *p* value less than 0.05 was considered significant.

First, to establish if Aq enhances the growth of *F. prausnitzii* we have grown the bacterium in the presence of 0.2, 0.5, 1 and 2% Aq and measured its growth through changes in optical density (O.D_600_) over a period of 24 h. As shown in Figure 1A, 0.2 and 0.5% Aq significantly stimulated (*p* < 0.05) the growth of *F. prausnitzii* in anaerobic conditions with a clear advantage at 0.5% concentration. Concentrations of 1 and 2% have also presented *F. prausnitzii* with a growth advantage over the untreated control but with no statistical significance. These results suggest that mixtures of natural antimicrobials can indeed enhance the growth of *F. prausnitzii* with maximum efficacy at concentrations of 0.5%. The effect of Aq on the relative abundance of bacteria containing the butyryl CoA transferase (*But*) gene (Figure 1B) indicates a possible increase in butyrate release in the supernatant. To further investigate this hypothesis, we have measured butyrate production by *F. prausnitzii* in minimal media (Figure 1C) over a period of 24 h (0, 3, 6, 12 and 24 h). Our results show that *F. prausnitzii* release of butyrate in the culture supernatants was significantly higher (*p* < 0.05%) in the presence of Aq when compared to the control. The peak of butyrate release (Figure 1C) was achieved at 0.5% Aq. Concentrations of 1 and 2% also show a significant increase in butyrate release compared to the control but not significantly different to the 0.5% concentration, indicating that a secretion plateau is being achieved above this concentration.

### 3.2. F. prausnitzii Growth and Butyrate Prodyction in a Shrimp Gut Model and an Irradiated Spiked Faeces Gut Model in the Presence of Aq

**Figure 2:**
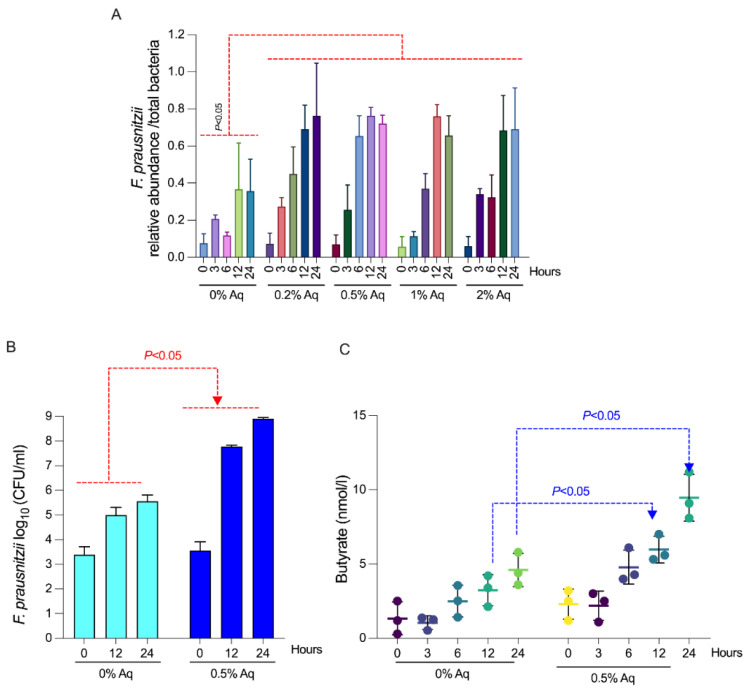
*F. prausnitzii* growth, relative to total bacteria as quantified by RT-PCR in an in vitro gut model with shrimp faeces. Panel (**A**) shows the ratio of *F. prausnitzii* abundance relative to total bacteria abundance. Panel (**B**) indicates the growth profile of *F. prausnitzii* in the presence of 0.5% Aq in an irradiated shrimp gut model. The graph shows the mean (±SEM; for time 12 and 24 h) log_10_ (CFU/mL) of the bacteria at 12 and 24 h of incubation with 0.5% Aq. Panel (**C**) presents the levels of butyrate released by *F. prausnitzii* in the irradiated shrimp gut model and the effect of 0.5% Aq. Butyrate production was measured at intervals of 0, 3, 6, 12 and 24 h. All experiments were performed triplicate and each measurement was performed from three separate samples. A *p* value less than 0.05 was considered significant.

Next, we aimed to investigate if Aq stimulates the growth of commensal *F. prausnitzii* in an in vitro shrimp gut model. The measurement performed at 0 h indicates the presence of *F. prausnitzii* as a commensal in the gut contents used in the in vitro model. As observed in Figure 2A, there was a significant effect (*p* < 0.05) on *F. prausnitzii* relative abundance at all concentrations of Aq when compared to control. All Aq concentrations improved *F. prausnitzii* relative abundance between 6–24 h of incubation in the gut model, with the highest impact being achieved at 0.5% Aq and no further increments at 1 or 2% Aq. These results indicate that Aq has the potential to increase the relative abundance of *F. prausnitzii* in the gut and improve gut health. In a *F. prausnitzii* spiked gut model, with irradiated faeces, there was a significant growth-promoting (*p* < 0.05) effect of Aq on *F. prausnitzii* between 12–24 h of incubation (Figure 2B). This increase in bacterial growth was also associated with a significant increment (*p* < 0.05) in butyrate formation between 12–24 h when compared to control (Figure 2C).

### 3.3. F. prausnitzii In Vitro Growth, Butyrate Production and Substrate Digestion

**Figure 3:**
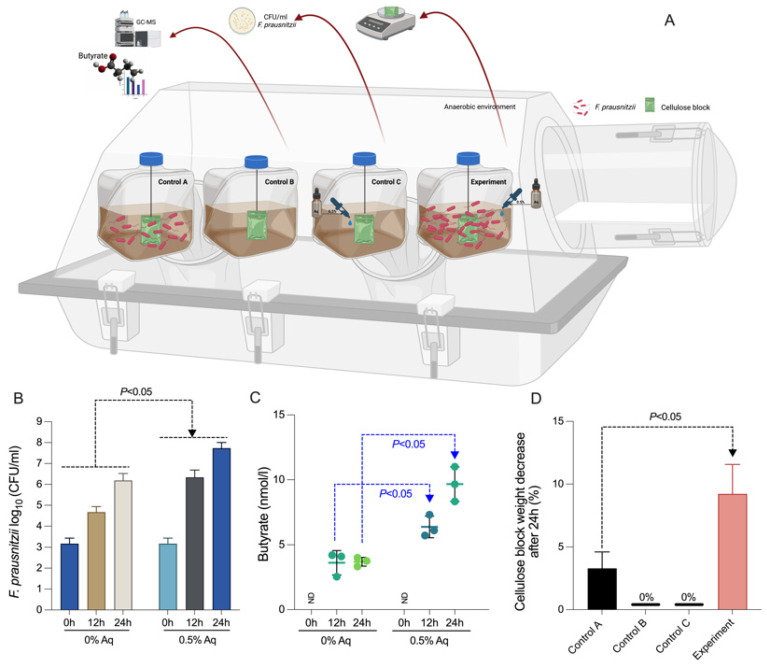
*F. prausnitzii* in vitro growth, butyrate production and substrate digestion. Panel (**A**)—experimental design (Created with Biorender.com), Panel (**B**)—*F. prausnitzii* growth, Panel (**C**)—butyrate production, Panel (**D**)—Cellulose degradation. All experiments were performed in triplicate. A *p* value of less than 0.05 was considered as significant.

To further connect the increase in butyrate production with the increase in bacterial growth, next, we aimed to demonstrate that these observations are based on increased substrate consumption. As shown in Figure 3A, we have used 3 controls (as described in Material and Methods) and one experimental group to test the effect of 0.5% Aq on the ability of *F. prausnitzii* to produce butyrate and use the available cellulolytic substrate. In Figure 3B, we show that at 0% Aq (Control A) *F. prausnitzii* growth is significantly slower (*p* < 0.05) when compared to the experimental group (Experiment) and in the presence of 0.5% Aq. Similarly, the same effect was observed when butyrate production was measured (Figure 3C). In the absence of *F. prausnitzii*, CFU and butyrate levels were not measured in control B and control C and are not represented in Figure 3B,C. Additionally, our data show (Figure 3D) that the increase in bacterial growth is not only associated with butyrate formation but also with an increase in substrate consumption. Over 9% of the substrate was consumed by *F. prausnitzii* within 24 h of incubation (Experiment) compared to less than 4% in Control A (*p* < 0.05). No change in the substrate was observed in controls B and C.

### 3.4. The Impact of Aq Grown F. prausnitzii on V. parahaeomolyticus A3 Infection of Primary Shrimp Gut Epithelial Cells (SGP)

**Figure 4:**
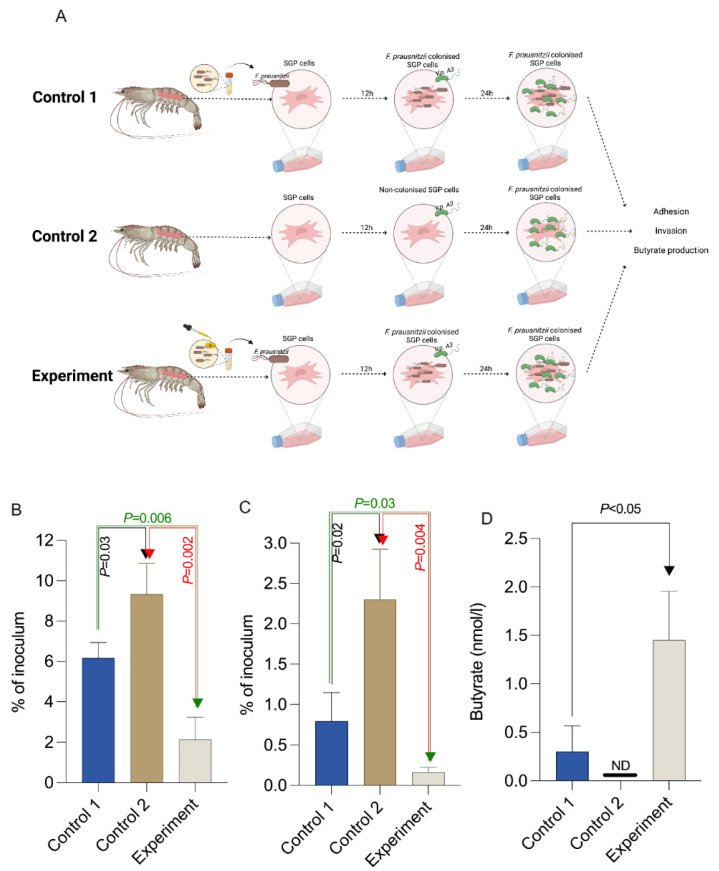
The effect of 0.5% Aq and *F. prausnitzii* in preventing *V. parahaeomolyticus* A3 infection of SGP cells. Panel (**A**)—Experimental design (Created with Biorender.com). Panel (**B**)—Adhesion to SGP cells by *V. parahaeomolyticus* A3; Panel (**C**)—Invasion of SGP cells by *V. parahaeomolyticus* A3. Panel (**D**)—Butyrate released in the culture supernatant during infection. The results are expressed as percentages of the initial inoculum. *p* values are indicated on the graphs (Student’s *t*-test) and the error bars represent the standard deviation of means from three different experiments, each containing triplicate samples.

This experiment was designed to investigate if *F. prausnitzii* exposure to Aq also impacts on its ability to reduce *V. parahaeomolyticus* A3 colonisation and infection of SGP cells. To avoid a direct effect of Aq on *V. parahaeomolyticus* A3 or on the SGP cells, *F. prausnitzii* were grown in the absence/presence of 0.5% Aq prior to SGP cells colonisation. The experimental design is described in material and methods and in Figure 4A. In the absence of 0.5% Aq (Control 1), *F. prausnitzii* is able to reduce the levels of *V. parahaeomolyticus* A3 attaching (Figure 4B) (*p* = 0.03) or invading SGP cells (Figure 4C) (*p* = 0.03) compared to the un-infected and un-treated control (Control 2). However, when SGP cells were colonised with *F. prausnitzii*, pre-exposed to 0.5% Aq, *V. parahaeomolyticus* A3 (Figure 4A, Experiment) adhesion (Figure 4B) (*p* = 0.002) or invasion (Figure 4C) (*p* = 0.004) levels were further reduced when compared to Control 2. Nevertheless, when we compared the infection levels of *V. parahaeomolyticus* A3 in the Experiment group with the levels observed in Control 1, there was a significant reduction in adherence (*p* = 0.003) and invasion (*p* = 0.004). Interestingly, Figure 4D also shows that there was a continuous significant (*p* < 0.05) release of butyric acid in the Experiment group. The concentration of 0.5% Aq was not cytotoxic to SGP cells. Taken together these results suggest that pre-exposure of *F. prausnitzii* to 0.5% Aq, prior to SGP cells colonisation, significantly prevents *V. parahaeomolyticus* A3 infection potentially due to the constant release in butyric acid in the supernatant.

## 4. Discussion

In shrimp, the presence of probiotic bacteria in the gut will stimulate growth, will improve immunity, and disease resistance and increase the activity of digestive enzymes [31]. Moreover, they will play a vital role in maintaining gut health through competitive pathogen exclusion or by producing other metabolites and increasing host resilience in disease scenarios [32]. In this study, we have identified that a mixture of organic acids was able to improve the growth and the relative abundance of probiotic bacteria, such as *F. prausnitzii*, both in in vitro cultures and in an in vitro gut model. Stimulation of probiotic bacteria abundance improves gut health in shrimp and, among other bacterial taxa, *F. prausnitzii* appears to be particularly increased in the healthy shrimp gut microbiota (*L. vannamei*), compared to the microbiota of shrimp affected by the acute hepatopancreatic necrosis disease (APHND) as a result of *V. parahaemolyticus* infection [11]. In our study, when SGP cells were colonised with *F. prausnitzii*, grown in the presence of 0.5% Aq, they were significantly less prone to *V. parahaemolyticus* adherence and infection. However, it is unclear what is causing the observed decrease in *V. parahaemolyticus* virulence, nevertheless we postulate that the increase in butyric acid released by Aq pre-exposed *F. prausnitzii* might be responsible. It is indeed possible for butyrate to cause such an effect since it has been shown as effective against *V. parahaemolyticus* biofilms [33]. Moreover, the role of butyric acid as an energy supplier and an enhancer of the epithelial barrier could offer an explanation for the observed reduction in *V. parahaemolyticus* infection levels in SGP cells [29].

Short-chain fatty acids (SCFAs) undoubtedly play an important role in the maintenance of health and the development of disease and they are mainly produced by the gut microbiota [34]. Mixtures of natural antimicrobials (AuraShield L) have been previously shown to increase SCFAs production in a viral chicken infection model alleviating the severity of disease, however, the source of SCFAs release was not identified [17]. However, *F. prausnitzii* has been shown to produce butyrate resulting in an anti-inflammatory role by inhibiting interleukin (IL-6), the activator of transcription 3 (STAT3)/IL-17 pathway in colorectal colitis having an important role in inflammatory bowel disease (IBD) treatment [35]. The production of butyrate by *F. prausnitzii* is a common feature in many isolates [36] and its production is correlated to its growth [37]. Our study also points out that the growth of *F. prausnitzii* correlates with increased butyrate production which is further enhanced in the presence of various concentrations of the organic acid mixture (Aq). Similar increments of SCFAs released by probiotic bacteria were reported in chitosan fed shrimp (*L. vannamei*) having a positive impact on the immune system and growth performance [38]. The impact of butyrate on performance and gut health was also suggested in an earlier study when the positive impact on intestinal morphology lead to improved digestive capacity of *L. vannamei* [39]. Moreover, Aq was indeed shown to have a positive impact on the performance, immune system, and gut health of *V. vannamei* in vivo [18]. Our study suggests that *F. prausnitzii* in one of the butyrate sources in the shrimp gut which is enhanced in the presence of Aq. One of the components of Aq, maltodextrin, indeed was shown to act as prebiotic and increases butyrate production in gut bacteria [40]. *F. prausnitzii* is certainly considered to be highly dependent on specific nutrients with and specific prebiotics or carbon sources have been used for to increase its abundance and promote gut health [41]. Prebiotics are indeed known for their role as substrate for probiotic-like bacteria, leading to increased production of host beneficial metabolites [42]. Our results also show, in a gut model, that *F. prausnitzii* itself can be responsible for such an increase in butyrate production in irradiated shrimp faeces by taking advantage of the Aq growth-promoting effect on *F. prausnitzii*. Other prebiotics were actually also shown to improve the growth of *F. prausnitzii* without affecting other gastrointestinal microorganisms, indicating a potential targeting effect [43]. As such, we speculate that due to the organic acid-based composition, Aq can have growth-promoting effects serving as an additional carbon source for *F. prausnitzii* nutritive requirements.

Butyrate is produced in the gut by many bacteria, including *F. prausnitzii*, through gastrointestinal fermentation of carbohydrates escaping digestion in the gut lumen [44]. This bacterium can decompose undigested cellulose in the intestinal lumen to produce butyrate, an important metabolite which improves the stability of the gut ecosystem [45]. Increasing cellulolytic activity in the shrimp gut, through the addition of the cellulolytic bacterium *Bacillus* sp. DDKRC1, increased feed efficiency and shrimp health and survival [46]. In the human gut, *F. prausnitzii*, can also contribute to the digestibility of various hemicelluloses derived from the primary mannanolytic activity of nearby gut microbiota [47]. We were able to further prove the connection between *F. prausnitzii* growth increase, promoted by Aq, and the increase in substrate digestion by using in vitro models. Additionally, this interconnection led to increased butyrate release in the culture supernatant. Conclusively, mixtures of organic acids, can lead to pathogen exclusion [14,15,16,18,48,49] by promoting the growth of probiotic-like bacteria and can stimulate the production of SCFAs with a health-beneficial effect on the host.

## 5. Conclusions

Our study shows that a mixture of organic acids (Aq) was able to stimulate the growth of *F. prausnitzii* in vitro culture and in an ex vivo shrimp gut model. The organic acid mixture stimulates increased production of butyrate in both models and is linked to a higher level of substrate utilization. By using an in vitro infection model, we have also shown that Aq-grown *F. prausnitzii* are able to reduce *V. parahaemolyticus* infection of shrimp primary gut cells while maintaining an increased production of butyrate. The results presented in this study improves our knowledge of how the growth and activity of host commensal probiotic-like bacteria can be improved to tackle specific infections in an aquatic environment where direct probiotic supplementation is difficult to implement.

## Data Availability

Not applicable.
